# *Mycobacterium xenopi* systemic infection in a domestic fiery-shouldered conure bird (*Pyrrhura egregia*)

**DOI:** 10.1099/jmmcr.0.005158

**Published:** 2018-07-04

**Authors:** Guillaume St-Jean, Carl A. Gagnon, Hafid Soualhine, Manon Tremblay, Andrée-Anne Beaulieu, Doris Sylvestre

**Affiliations:** ^1^​ Département de pathologie et microbiologie, Faculté de médecine vétérinaire, Université de Montréal, St-Hyacinthe, Canada; ^2^​ Swine and Poultry Infectious Diseases Research Centre (CRIPA), Faculté de médecine vétérinaire, Université de Montréal, St-Hyacinthe, Canada; ^3^​ Laboratoire de Santé Publique du Québec, Sainte-Anne-de-Bellevue, Canada; ^4^​ Service Vétérinaire à Domicile pour Oiseaux et Animaux Exotiques, Montréal, Canada; ^5^​ Département de microbiologie, Hôpital Honoré-Mercier, St-Hyacinthe, Canada; ^6^​ Laboratoire de santé animale (LSA-St-Hyacinthe), Ministère de l'Agriculture des Pêcheries et de l'Alimentation du Québec, Canada

**Keywords:** *Mycobacterium xenopi*, mycobacterium infection, Avian, granulomatous

## Abstract

**Introduction:**

*Mycobacterium xenopi* is a rare opportunistic pathogen mainly causing infections in immunocompromised human patients or those with underlying chronic structural lung disease. Cases of disease in veterinary medicine remain scarce. Few animal species, including birds, are suspected of being vectors of the disease and there has not yet been a report of clinical disease in birds. We report the first case, to our knowledge, of systemic infection in a domestic bird.

**Case presentation:**

A female fiery-shouldered conure was submitted after death for necropsy following episodes of heavy breathing. The necropsy revealed multiple granulomatous lesions within the liver, air sacs and kidneys. Ziehl–Neelsen stains demonstrated the presence of numerous intralesional acid-fast bacilli. PCR assays and culture confirmed the presence of *M. xenopi*.

**Conclusion:**

Through this case we hope to describe the characteristics of *M. xenopi* disease in birds and the possible close relationship between animal and human infections.

## Introduction

Mycobacteria are aerobic, non-motile, acid-fast bacteria from the family *Mycobacteriaceae*. There are more than 150 recognized species of mycobacteria, which can be further classified based on additional characteristics, including growth rate, growth substrate and pigmentation. In medicine, mycobacteria are mostly classified by the pathogenic characteristics of individual species [1]. In humans, the main pathogens of concern belong to the *Mycobacterium tuberculosis* complex, which includes the causative agents of human tuberculosis and leprosy (*Mycobacterium tuberculosis, Mycobacterium bovis, Mycobacterium africanum, Mycobacterium caprae, Mycobacterium microti, Mycobacterium leprae* etc.). Nontuberculous (NTM) or atypical mycobacteria include all other environmental or pathogenic mycobacteria and several of them are considered opportunistic pathogens. They may be the source of important diseases in humans (mainly pulmonary, cutaneous and disseminated) depending on the route of infection, immune status of the host and pathogenicity of the species involved [2]. *Mycobacterium xenopi* is a slow-growing, nontuberculous mycobacteria first isolated from granulomatous skin lesions on toads. The natural reservoir of the bacteria is water. It has been isolated on occasion in wild birds with no signs of disease, which indicates that they could be a possible source of environmental contamination [3]. We report the first case, to our knowledge, of systemic infection in a domestic bird. Through this case we hope to describe the characteristics of *M. xenopi* disease in birds and the possible close relationship between animal and human infections.

## Case report

A 4 year-old female fiery-shouldered conure (*Pyrrhura egregia*) was submitted to the diagnostic service at the Faculté de médecine vétérinaire for post mortem examination in September 2015. The animal was bought in December 2014 from a breeder located in Ontario, Canada. The animal travelled by air to Montréal, Québec, Canada, where quarantine was established for 30 days. The new owner noted brittle feathers, but the overall behavior was normal. The new owner’s farm consisted of breeding pairs from different species (conures, amazon parakeets). The animal was housed in a controlled environment with adequate ventilation and temperatures. Cages were cleaned every 4 days with a mixture of water and vinegar. Recycled paper was used as litter. Birds had unlimited access to water and dry food (Roudy Bush). Couscous, fresh fruits and fresh vegetables were available 5 h a day. The animal was newly introduced in the owner’s farm with a male from the same species previously purchased in Québec, Canada. No breeding was reported before death. Overall, the animal health surveillance program was minimal and consisted mainly of weighing the animal twice a year. No significant weight loss of the bird was reported prior to its sickness. The owner reported that the animal was drowsy and presented labored breathing when resting over a period of one week and it died before its physical examination could be conducted or a blood sample could be collected.

**Fig. 1.**
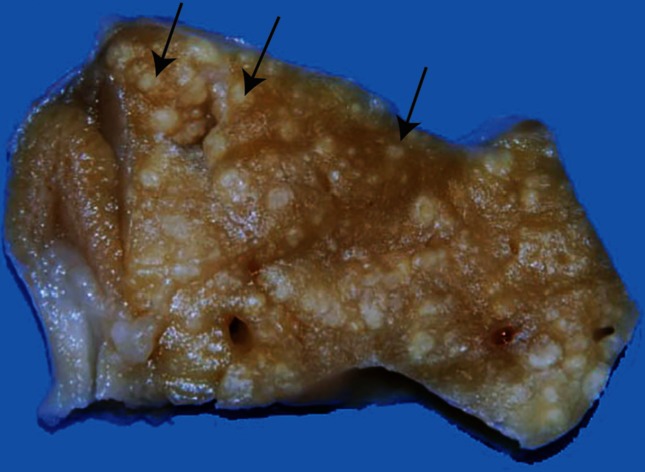
*Mycobacterium xenopi* infection in the liver of a bird. The liver contains numerous randomly distributed and irregular white nodules throughout the parenchyma (black arrows). Macroscopic.

The animal’s body was in good condition with ruffled feathers on the head, back and distal third of the wings. In the coelomic cavity, the liver was enlarged, with irregular edges and multiple adhesions to the coelomic wall. The liver contained numerous irregular, slightly elevated, white nodules measuring between 0.1 and 0.3 cm in diameter, which were randomly distributed in the parenchyma (Fig. 1). Similar nodules were observed at the surface of the air sacs. The lungs and kidneys were congested.

**Fig. 2.**
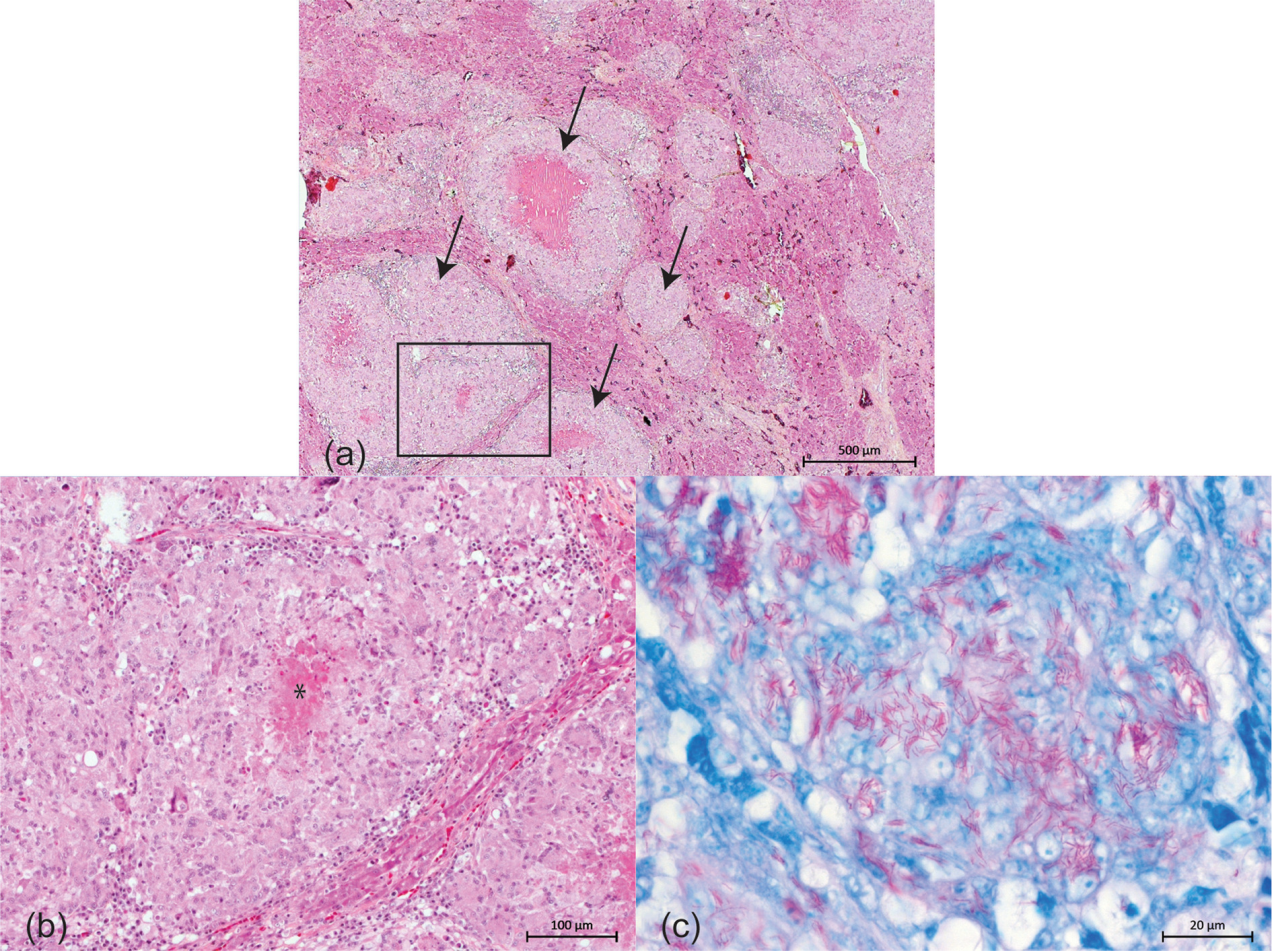
*Mycobacterium xenopi* infection in the liver of a bird. (a) The liver parenchyma is infiltrated by large amounts of macrophages and multinucleated giant cells forming large granulomas (black arrows). Hematoxylin phloxin saffron (HPS). Bar, 500 µm. (b) Higher magnification from the framed section of Fig. 2(a). The center of numerous granulomas (*) is often necrotic and adjacent parenchyma compressed. HPS. Bar, 100 µm. (c) The granulomas contain large amounts of acid-fast bacteria which are mainly observed in the cytoplasm of macrophages and multinucleated giant cells. Ziehl–Neelsen. Bar, 20 µm.

Tissues were fixed in 10 % buffered formalin, paraffin embedded and cut at 3 µm thickness for microscopic evaluation. The liver was severely infiltrated by macrophages and multinucleated giant cells, forming numerous round, irregular foci which were often centered around eosinophilic material and cellular debris (granulomas) (Fig. 2a, b). The macrophages were large with abundant eosinophilic cytoplasm. There was a mild and multifocal periportal fibrosis with mild biliary duct hyperplasia and lymphohistiocytic infiltration. The liver capsule was slightly edematous and thickened by fibrin. Ziehl–Neelsen staining revealed the presence of numerous intracytoplasmic, acid-fast bacilli in the macrophages and multinucleated giant cells (Fig. 2c). Similar granulomas were also observed in air sacs, kidneys and, rarely, in the vascular walls. Additional lesions included lymphohistiocytic infiltration of the pericardium and peribronchiolar tissue.

Liver tissue was submitted to the molecular diagnostic laboratory of the Faculté de médecine vétérinaire of Université de Montréal for further characterisation of the mycobacterium specimen. First, DNA was extracted from a liver tissue homogenate using the QIAamp DNA mini kit (Qiagen) with the Qiacube apparatus following the manufacturer’s instructions. Thereafter, a polymerase chain reaction (PCR) assay was conducted based on an adapted protocol from Hashimoto *et al*. which was designed to sequence the 16S ribosomal RNA gene, allowing for the identification of a broad range of mycobacterial species [4]. The obtained PCR amplicon was sequenced and this sequence was submitted to GenBank’s Basic Local Alignment Search Tool (blast) for comparison. The sequence demonstrated 100 % homology to *M. xenopi* sequences.

Tissue samples (liver, kidneys and lungs) were submitted for routine bacterial culture. The samples were cultivated on Columbia agar 5 % sheep blood at 35±2 °C with 5 % CO_2_ to evaluate the possibility of a bacterial infection. Fungal culture was also performed on Sabouraud agar at 30 °C for 21 days. No growth was observed overall.

Frozen sections of the liver, lungs and digestive tract were also submitted to the Laboratoire de Santé Publique du Québec (LSPQ) for mycobacterial isolation to confirm the presence of *M. xenopi* in the affected lesions. Liver and lung samples were processed at Hôpital Honoré-Mercier’s microbiology laboratory in St-Hyacinthe prior to submission to the LSPQ. Ground pieces of digestive tract (3 ml) were incubated with 0.9 % hexadecylpyridinium chloride (HPC) (Sigma Chemical) at 37 °C for 24 h and then centrifuged at 900 ***g*** for 30 min. The pellets were collected and re-suspended in sterile water (1 ml). Liver, lung and treated digestive tract samples were incubated in liquid Bactec MGIT 960 system (Becton Dickinson Microbiology Systems) and solid Middlebrook 7H10 agar and Löwenstein–Jensen medium.

Pure and confluent mycobacterial cultures were obtained for the liver and digestive tract samples. Mixed culture was observed for lung tissue containing slow-growing mycobacteria with few Gram-negative bacteria. DNA was extracted using the BioRobot M48 (Qiagen) as per laboratory protocol. Identification was performed by 16S ribosomal RNA (rRNA) gene sequencing. Sequence alignment has shown a 100 % identity with the sequence of the type strain *M. xenopi* ATCC 19250.

The final diagnosis was consistent with disseminated avian mycobacteriosis due to *M. xenopi*.

In the four months following the diagnosis, four other birds from the owner’s farm that had died were sampled. PCR assays were conducted on different samples (liver, digestive tract, lungs) for mycobacteria and these were negative.

## Discussion

In veterinary medicine, specifically in mammals, the most notable mycobacteria are *M. bovis* and *M. paratuberculosis. M. bovis* is mostly observed in ruminants and is the cause of bovine tuberculosis; however, it can affect a wide range of domestic species, including pigs, goats, camelids, horses, cats and dogs [5], and wild animals [6]. Lesions consist of granulomatous diseases mostly affecting the respiratory system [7]. Clinical tuberculosis is rare in domestic Canadian animals, but still occurs in regions where bovine herds may be in contact with infected wildlife (bovids and cervids) [8, 9]. In Canada, *M. bovis* is a reportable disease due to its zoonotic potential. *M. paratuberculosis* also causes granulomatous diseases in ruminants. The most common form of paratuberculosis diseases is characterized by diffuse granulomatous enteritis and/or lymphadenitis in young animals. The infected animals can shed *M. paratuberculosis* into the environment through feces [10].

In birds, most cases of mycobacteriosis are caused by *Mycobacterium avium* sp. *avium* and *Mycobacterium genavense* with rare occurrences of other mycobacteria (including *M. bovis)*. Both mycobacteria may affect a wide range of domestic and wild birds including chickens and turkeys. Thus, mycobacterial infections may cause important economic losses in commercial flocks. Avian disease is characterized by systemic granulomatous lesions. The primary source of infection remains environmental, and infected birds can shed the bacteria in feces via ulcerated lesions in the digestive tract. Susceptible birds may be infected via ingestion or inhalation of infectious organisms. The bacteria may survive in the soil for years, and this can lead to the re-emergence of disease in new birds and poses an important concern for domestic bird breeders [11]. Stress factors, such as manipulations and introduction of new animals, contributes to the susceptibility of birds to the disease.

*M. xenopi* infections are of increasing interest in human medicine due to the emergence of nosocomial pulmonary infections, which have been associated with contamination of drinking water systems [12–15]. The infection is mostly observed in immunocompromised patients, in which *M. xenopi* can cause a wide range of granulomatous lesions in the lung, bones and other tissues [13, 16–18]. Pulmonary lesions may be similar to those encountered in tuberculosis, which emphasises the importance of a precise diagnosis. Emerging cases have also been reported in non-immunocompromised patients affected by pulmonary diseases such as chronic obstructive pulmonary disease [19].

The disease remains rare in veterinary medicine. Reported cases include skin lesions in toads, lymphadenitis in pigs and cutaneous or systemic lesions in cats and ferrets [20–23]. We present the first clinical case, to our knowledge, of a *M. xenopi* systemic infection in a bird (*Pyrrhura egregia*). The diseases associated with *M. xenopi* in veterinary medicine are similar to those observed with other non-tuberculous mycobacteria and indicate that immunocompromised animals are more susceptible to the disease. For instance, a specific case of systemic infection of similar severity to our case was reported in a cat suffering from idiopathic CD4+ T lymphopenia [20, 24]. In our case, there were no evidence of other simultaneous infectious diseases. However, other stressful events, such as manipulations, relocation of the animal from Ontario to Québec and introduction of other animals, may have contributed to the development of the disease.

While the source of infection in our case remains undetermined, water contamination may be considered highly probable as it has been reported as a natural reservoir. For a recent case reported in an albino ferret it was possible to associate the source of infection with contaminated water from an aquarium to which the animal was exposed [23]. It is interesting to note that the province of Ontario, where the animal comes from, has been experiencing a significant increase in human infections due to *M. xenopi* [12]. Recent measures indicate that *M. xenopi* is the second most prevalent nontuberculous mycobacterial disease in the province of Ontario, after the *M. avium* complex [25]. Thus, there may be an increased prevalence of environmental contamination in this geographical region and this is presumably where the bird’s infection started. As mentioned before, no other animals at the owner’s farm suffered from mycobacteriosis in the month following this case. However, due to limitations, the environment could not be sampled, and we cannot determinate whether the bird could have been a source of environmental contamination.

This case depicts the unique nature of *M. xenopi* infection, indicating that birds are also susceptible to *M. xenopi* infections and should not be considered only as reservoirs. Furthermore, the geographical prevalence of the disease indicates that animals and humans may show similar susceptibility regarding the exposure to a common contaminated environment. Thus, care should be taken when animals are moved from possibly contaminated regions, as they may be infected or contaminate their new environment.
